# Impact of hospice care plus estazolam on sleep and psychological distress in advanced gastrointestinal cancer: a real-world observational study

**DOI:** 10.3389/fmed.2026.1827918

**Published:** 2026-06-03

**Authors:** Lihong Hu, Donghui Cao, Xiaoya Yuan, Xiangtuan Wang, Haifeng Yuan

**Affiliations:** 1 Department of Geriatrics and Specialized Medical Care, General Hospital of Ningxia Medical University, Ningxia Hui Autonomous Region, Yinchuan, China; 2 Department of Spinal Orthopedics, General Hospital of Ningxia Medical University, Yinchuan, China; 3 The First Clinical College of Ningxia Medical University, Ningxia Hui Autonomous Region, Yinchuan, China; 4 University of Southern California, Los Angeles, CA, United States

**Keywords:** advanced gastrointestinal cancer, estazolam, hospice care, psychological state, sleep quality

## Abstract

**Objective:**

To retrospectively evaluate the effects of hospice care combined with estazolam on sleep quality and psychological status in patients with advanced gastrointestinal cancer.

**Methods:**

In accordance with our predefined inclusion and exclusion criteria, this study retrospectively enrolled 110 inpatients with advanced gastrointestinal cancer and sleep disturbances who received treatment at our hospital between January 2019 and January 2025. Participants were assigned to two groups based on the treatment documented in their medical records: the hospice care plus estazolam group (*n* = 59) and the routine nursing plus estazolam group (*n* = 51). The main evaluation indicators include changes in sleep quality, the degree of anxiety and depression, which were assessed using the Pittsburgh Sleep Quality Index (PSQI), the Self-Rating Anxiety Scale (SAS), and the Self-Rating Depression Scale (SDS), respectively. The secondary evaluation indicators include quality of life and symptom burden, which were assessed using the European Organization for Research and Treatment of Cancer Quality of Life Questionnaire (QLQ-C30) and the Edmonton Symptom Assessment Scale (ESAS), respectively. Statistical analysis was performed using SPSS software. Relevant scoring indicators were compared between the two groups at baseline and 1 month after the initiation of treatment. The chi-square test and *t*-test were used to analyze intergroup differences. Adverse events were recorded and compared between groups.

**Results:**

After 1 month of treatment, the hospice-care plus estazolam group showed greater improvements than the routine nursing plus estazolam group: PSQI (sleep quality) mean difference between groups was significant (*P* < 0.001, Cohen’s *d* = 1.043); anxiety (SAS) *P* < 0.001, *d* = 1.10; depression (SDS) *P* < 0.001, *d* = 1.15—all reflecting large effect sizes. The ESAS appetite subscale yielded a Cohen’s *d* of 0.758, indicating a moderate-to-large effect, while all other ESAS and QLQ-C30 subscales had Cohen’s d values above 1.0, also indicating large effects. These findings suggest that the combined treatment was associated with substantial clinical improvements in sleep, psychological state, and overall quality of life.

**Conclusion:**

This retrospective analysis suggests that hospice care combined with estazolam is associated with significantly better sleep quality, psychological wellbeing, and overall quality of life in patients with advanced gastrointestinal cancer compared to routine nursing with estazolam alone. However, due to the retrospective design and short 1-month follow-up, causality cannot be inferred, and the study has limitations including a relatively small sample size, single-center design, and short follow-up duration. Future prospective studies with larger samples and longer follow-up are needed to confirm these findings.

## Introduction

Patients with advanced gastrointestinal cancer often face a range of complex symptoms, with sleep disturbances and psychological distress being particularly prominent (1, 2). These issues are commonly linked to disease progression, chronic pain, anxiety, and the fear of death (3). Studies have shown that approximately 70% of patients with advanced cancer experience varying degrees of sleep disturbances, often accompanied by psychological issues such as anxiety and depression (4).

In patients with advanced cancer experiencing insomnia, pharmacological treatments such as benzodiazepines, sedative-hypnotic medications, and low-dose antidepressants are commonly utilized to alleviate sleep disturbances and associated anxiety symptoms (5, 6). Estazolam—a commonly prescribed benzodiazepine—possesses an intermediate elimination half-life of approximately 10–24 h, compared to much shorter-acting agents such as triazolam. This longer duration enables patients to maintain sleep throughout the night and helps prevent early morning awakenings—an especially valuable property for individuals with advanced cancer. Its dual pharmacologic actions—facilitating both hypnotic and anxiolytic effects—make it particularly beneficial for this population, who frequently suffer from concurrent insomnia and anxiety (7). Furthermore, animal studies have not shown carcinogenic effects, and there is no evidence of human carcinogenic risk. Its side effect profile is generally manageable (8). However, monotherapy with estazolam may lead to tolerance and dependence, and long-term use could increase the risk of adverse reactions (9). This is particularly concerning for frail or advanced cancer patients, as it may further compromise their physical and mental health.

Non-pharmacological interventions, such as cognitive-behavioral therapy for insomnia (CBT-I), sleep hygiene measures, mindfulness-based interventions, bright light therapy, and exercise modalities, have shown therapeutic promise (10). A scoping review by Munir et al. (11) reported that, although many individual studies lacked statistical significance, patients with advanced cancer experienced sleep improvements following CBT-I and similar approaches. Hospice care, as a comprehensive and patient-centered model, emphasizes holistic attention to the physical, psychological, social, and spiritual needs of terminal patients. It offers elements such as comfort-focused nursing, emotional support, family involvement, and social-resource linkage—areas that pharmacotherapy alone cannot address (12). Bakitas et al. (13) research has demonstrated that hospice care can effectively alleviate anxiety and depression, improve sleep quality, and enhance the overall quality of life for patients with advanced canc. In addition, hospice provides a calm medical environment through systematic counseling, symptom management, and comfort care, reducing patients’ psychological burden and improving overall health (14). However, barriers such as small sample sizes, mixed results, limited accessibility of trained therapists, and feasibility challenges in palliative settings hinder widespread adoption. These factors underscore the ongoing need for integrative and adaptable treatment models.

Most previous research has combined hospice care with medications for symptom management—such as analgesics, sedatives, anxiolytics, antipsychotics, and anticholinergics—for patients with advanced cancer (15–17). However, few studies have focused on sleep disturbances, a symptom that can gradually lead to significant psychiatric issues and diminish patient quality of life (18). Hospice care provides comprehensive support across physical, psychological, social, and spiritual domains—offering depth and breadth that pharmacotherapy alone cannot deliver. When paired with estazolam, which delivers rapid hypnotic and anxiolytic effects, this integrated model enables both swift symptom relief and holistic comfort, potentially yielding synergistic outcomes. While benzodiazepines are frequently used in hospice for symptoms like insomnia and anxiety, no studies to date have systematically evaluated the combination of hospice care with estazolam. The research question of this retrospective study was: among patients with advanced gastrointestinal cancer and sleep disturbances (Population), is hospice care plus estazolam (Intervention) associated with greater improvements in sleep quality, psychological status, quality of life, and symptom burden compared with routine nursing plus estazolam (Comparison) over a 1-month period (Time frame)? Specifically, the primary outcomes were changes in sleep quality (measured by the Pittsburgh Sleep Quality Index, PSQI), anxiety (Self-Rating Anxiety Scale, SAS), and depression (Self-Rating Depression Scale, SDS). Secondary outcomes included quality of life (QLQ-C30) and symptom burden (Edmonton Symptom Assessment Scale, ESAS). The results may offer novel clinical strategies and theoretical foundations for integrative and individualized care in advanced cancer patients.

Hospice care in China differs from models in Western countries. In the United States and United Kingdom, hospice care is often community-based or home-based, with well-established integration into primary care. In contrast, hospice care in China is still an evolving concept and is predominantly hospital-based, delivered by multidisciplinary teams within tertiary hospitals. It emphasizes symptom control, psychological support, and family involvement, though standardization, reimbursement mechanisms, and accessibility remain challenges. Estazolam, an intermediate-acting benzodiazepine, remains in clinical use in China for insomnia and anxiety in palliative settings, partly due to its availability, low cost, and familiar safety profile. Understanding this local context is essential for interpreting our findings and for comparing results across different healthcare systems.

## Materials and methods

### Study design

This study was a retrospective cohort analysis. A retrospective design was chosen because randomizing patients to receive either hospice care or routine nursing is ethically and practically challenging in the terminally ill population. Furthermore, real-world observational data can provide valuable preliminary evidence for this understudied combination, informing the design of future prospective studies. According to predefined inclusion and exclusion criteria, between January 2019 and January 2025, we screened the medical records of inpatients with advanced gastrointestinal cancers complicated by sleep disturbances at the General Hospital of Ningxia Medical University. After excluding 63 patients who did not meet the criteria, 120 eligible patients were identified and divided into two groups based on the treatment they actually received as documented in their medical records: the hospice care combined with estazolam group (*n* = 60), and the routine nursing care combined with estazolam group (*n* = 60). During the follow-up period, one patient in the hospice care group and nine in the routine care group did not have complete post-treatment assessment data. Reasons for these exclusions were documented. Ultimately, 110 patients were analyzed—59 in the hospice care group and 51 in the routine care group. Baseline characteristics of both groups were compared accordingly:

- Hospice Care Group (*n* = 59): Patients received hospice-style palliative care combined with estazolam. Baseline characteristics were: 32 males and 27 females, mean age 60.68 ± 7.39 years, BMI 20.69 ± 1.42 kg/m^2^;
- Routine Care Group (*n* = 51): Patients received conventional nursing care combined with estazolam. Baseline characteristics were: 35 males and 16 females, mean age 58.53 ± 7.91 years, BMI 20.39 ± 1.08 kg/m^2^.

Baseline comparisons (Table 1) indicated no significant differences in sex, age, or BMI between groups (all *P* > 0.05), demonstrating that baseline characteristics were well-balanced. Ethical approval was obtained from the Ethics Committee of the General Hospital of Ningxia Medical University (Approval No. KYLL-2019-0921), and the study adhered to the principles of the Declaration of Helsinki. The requirement for written informed consent was waived due to the retrospective nature of the study.

**TABLE 1:** Baseline characteristics of patients (*n* = 110).

General information	Hospice care group (*n* = 59)	Routine care group (*n* = 51)	*t*/χ^2^	*P*
Age (years)	60.68 ± 7.39	58.53 ± 7.91	1.472^a^	0.144
Sex				
Male	32(54.24%)	35(68.63%)	2.379^b^	0.170
Female	27(45.76%)	16(31.37%)		
Body mass index (BMI, kg/m^2^)	20.69 ± 1.42	20.39 ± 1.08	1.241^a^	0.217
Tumor type				
Stomach cancer	5(8.47%)	7(13.73%)	6.603^b^	0.580
Gallbladder cancer	4(6.78%)	5(9.80%)		
Sigmoid cancer	7(11.86%)	8(15.69%)		
Rectal cancer	10(16.95%)	3(5.88%)		
Pancreatic cancer	8(13.56%)	5(9.80%)		
Liver cancer	12(20.34%)	11(21.57%)		
Esophageal cancer	5(8.47%)	7(13.73%)		
Appendix adenocarcinoma	4(6.78%)	4(7.84%)		
Bile duct cancer	4(6.78%)	1(1.96%)		
ECOG PS (0–2/3–4)	46/13 (78.0%/22.0%)	37/14 (72.5%/27.5%)	0.425^b^	0.514
Pain NRS	5.32 ± 1.45	5.51 ± 1.38	0.797^a^	0.428

^a^Independent samples *t*-test.

^b^Chi-square (χ^2^) test. Data for ECOG PS were missing for 4 patients (2 in hospice group, 2 in routine group); pain NRS was recorded for all patients at admission.

To further examine baseline comparability, we extracted documented ECOG performance status (PS) and pain Numerical Rating Scale (NRS) scores from medical records where available (ECOG PS: 96.4% of patients; pain NRS: 100%). Other important confounders, such as detailed opioid consumption, nutritional status (albumin/prealbumin), prior psychiatric medication, and baseline palliative care involvement, could not be reliably retrieved due to inconsistent documentation in retrospective data.

### Inclusion criteria

(1) Diagnosed with advanced gastrointestinal cancer, confirmed as advanced cancer based on clinical and pathological diagnostic criteria, with an expected survival period of more than 3 months but less than 6 months;
(2) All patients included in the study must exhibit varying degrees of sleep disturbances (such as difficulty falling asleep, early awakening, nocturnal awakenings, etc.), with a Pittsburgh Sleep Quality Index (PSQI) score of ≥ 10, confirming the presence of significant sleep issues (19);
(3) All patients enrolled in the study met the criteria for clinical significance on either the SAS or SDS scales: specifically, an SAS index score of ≥ 45 indicated the presence of anxiety (20), while an SDS index score of ≥ 53 indicated the presence of depression (21).

### Exclusion criteria

(1) Comorbid serious physical illnesses (e.g., liver or kidney failure, serious cardiovascular disease, etc.), which may directly affect the accuracy of the treatment results and increase the risk of treatment for the patient;
(2) patients with a history of previous psychiatric disorders (e.g., depression, anxiety disorders, schizophrenia, etc.), which may impair the effectiveness of participation in hospice and medication in the absence of relevant medications and treatments;
(3) patients with a history of allergy to benzodiazepines to avoid unwanted side effects due to drug allergy;
(4) patients who are unable to cooperate in completing the completion of assessment tools or interventions due to unconsciousness, cognitive impairment, etc.;
(5) patients with advanced malignant tumors metastasized to other organs, resulting in the inability of the patient to participate in the complete treatment and assessment.

### Sample size considerations

Due to the lack of preliminary data on expected effect sizes and variability specific to the combined hospice care and estazolam treatment in late-stage gastrointestinal cancer, no formal a priori sample size calculation was performed. Instead, our chosen sample size of 110 patients was based on logistical feasibility and comparisons with similar palliative care studies, which range from 50 to over 200 participants and often yield statistically significant findings. To retrospectively assess the adequacy of our sample size, we applied Lehr’s rule of thumb, a widely used estimate for two-group comparisons aiming for 80% power at a 0.05 alpha level:


$$n_{p\,e\,r\,g\,r\,o\,u\,p}\approx \frac{16}{\Delta^{2}}$$

where Δ denotes the standardized effect size (Cohen’s *d*) (22). Given our observed effect sizes (Cohen’s *d* ≈ 0.8–1.2), the required per-group sample size would range from approximately 16 (for *d* = 1.0) to 40 (for *d* = 0.6), indicating that our total sample of 110 patients is sufficiently powered to detect moderate to large effects.

### Treatment measures

#### Estazolam dosage

Both groups followed the “start with low dose and titrate slowly” strategy, with the specific dosage regimen referred to in the 19th edition of the Merck Manual. For adult patients with insomnia, the starting dose was set at 1 mg orally every night, with a target dosage range of 1–2 mg, and a single dose should not exceed 2 mg/day. In special cases, a multidisciplinary consultation and evaluation were required. For elderly patients aged 65 years and above, the starting dose was 0.5 mg/day, with a target dosage range of 0.5–1 mg/day, and the maximum dose was limited to 1 mg/day. During dose adjustment, decisions were individualized based on several factors—the degree of reduction in sleep onset latency (measured by PSQI), patient tolerability, hepatic and renal function (evaluated via eGFR and Child–Pugh score), and central nervous system side effects (such as dizziness, next-day somnolence, ataxia, or respiratory depression). In addition, dynamic safety monitoring points were set (baseline, day 7, day 14, and day 28). Patients with respiratory depression (SpO_2_ < 92%) or liver dysfunction (ALT/AST increased to more than twice the normal upper limit) were subjected to mandatory dose reduction or discontinuation. All dose adjustments were performed by a clinical pharmacy team using a standardized assessment form, with detailed documentation of rationale and patient response to ensure a consistent, reproducible dosage titration process.

#### Routine care group

Patients whose records indicated they received routine care combined with oral estazolam treatment. Routine Care Measures: i. Basic Care: Maintain a quiet and clean hospital environment, ensuring patient comfort, avoiding external noise interference, and providing good conditions for rest; ii. Dietary Guidance: Develop an individualized diet plan based on the patient’s condition and digestive function, encouraging the intake of high-protein, high-calorie, easily digestible foods to maintain nutritional status; iii. Symptom Management: Administer symptomatic supportive treatment as prescribed, such as analgesics, antiemetics, and digestive aids, to alleviate patient discomfort; iv. Health Education: Provide regular education to patients and their families on disease knowledge, medication precautions, and lifestyle care guidance to enhance patient compliance; v. Psychological Support: Basic psychological care, actively listening to patient concerns and promptly addressing negative emotions, but no systematic psychological intervention was implemented.

#### Hospice care group

Patients whose records indicated they received hospice care combined with oral estazolam treatment. In addition to the routine care provided to the routine care group, comprehensive hospice care interventions were documented: (1) Hospice Care Team: A dedicated hospice care team was formed, consisting of a senior oncologic surgeon, professional nurses, a psychological counselor, social workers, nutritionists, and hospice care experts. All team members underwent professional hospice care training to ensure comprehensive support for patients on physical, psychological, social, and spiritual levels; Use a structured intervention checklist to record key measures. The research team regularly reviewed medical documents to ensure consistency in protocol implementation, and maintained quality control through regular case discussions (23). (2) Hospice Care: i. Physical Care: Comfort care measures were implemented, such as regular repositioning, massage, and maintaining clean and dry bed linens to prevent pressure ulcers and aspiration pneumonia. Scientific medication, pain management, nutritional support, and symptom control were used to improve patient comfort and alleviate physical symptoms. ii. Psychological Care: The patient’s psychological status was assessed, and an individualized psychological counseling plan was developed. Methods such as communication, active listening, and empathy were used to help patients express their emotions and alleviate anxiety, fear, and depression. If necessary, a psychological counselor or clinical psychologist was involved in the intervention. iii. Social Support: Family members were encouraged to participate in the care process and were guided on how to provide effective companionship and emotional support. A warm and safe environment was created for the patient to enhance the emotional support from family and society. iv. End-of-Life Care (24, 25): For patients in the terminal phase, end-of-life care was provided, respecting the patient’s wishes and helping them alleviate suffering while ensuring a dignified end-of-life experience (Table 2).

**TABLE 2:** Structured hospice care intervention protocol.

Component	Frequency/Duration	Responsible staff	Documentation
Psychological counseling	30 min, twice weekly	Licensed psychologist	Session notes
Social support (family involvement)	30 min, daily	Social worker/nurse	Family meeting log
Comfort care (massage, repositioning)	15–20 min, twice daily	Trained nurse	Daily care checklist
Symptom management (pain, nausea, dyspnea)	As needed, reviewed daily	Palliative care physician	Medication and ESAS record
Environmental optimization (noise/light control)	Continuous	Nursing staff	Environmental rounds log
Nutritional support	Assessed weekly; 30 min counseling	Dietitian	Nutritional assessment form
End-of-life care planning	60 min, once (or as needed)	Hospice team leader	Advance care directive

To ensure intervention fidelity, all team members received standardized training. A structured intervention checklist was used to record delivery of each component. Adherence was defined as completion of ≥ 80% of planned components per patient per week. Weekly case discussions were held to review adherence and address deviations. Fidelity monitoring was performed by an independent study coordinator who reviewed 20% of randomly selected records; inter-rater agreement was 94%. In the hospice care group, mean adherence to the protocol was 91.3% (range 82–100%). No significant differences in adherence were observed across patient subgroups.

### Observation indicators and evaluation criteria

The main evaluation indicators in this study include changes in sleep quality, the degree of anxiety and depression, which were assessed using the PSQI (26), SAS (20), and SDS (27), respectively. The secondary evaluation indicators included quality of life and symptom burden, which were assessed using the QLQ-C30 (28) and ESAS (29), respectively.

All of these instruments have been validated for use in Chinese populations. Specifically, the Chinese version of the PSQI has demonstrated good reliability (Cronbach’s α = 0.82–0.90) in Chinese cancer patients; the SAS and SDS are widely used in Chinese clinical settings with acceptable psychometric properties; the QLQ-C30 has been validated in Chinese cancer populations; and the ESAS has been culturally adapted and used in Chinese palliative care settings.

Participants were evaluated consistently at both baseline and 1-month follow-up using the same assessment tools—PSQI, SAS, SDS, QLQ-C30, and ESAS—conducted at the same time and location for each individual. This uniform timing and setting ensured comparability across groups and minimized bias due to temporal variability. The primary goal was to evaluate changes between groups over time to comprehensively assess the treatment outcomes in improving sleep quality, alleviating symptom burden, and enhancing psychological wellbeing. Specifically:

1. The PSQI total score ranges from 0 to 21 points, with higher scores indicating poorer sleep quality. As a self-reported measure, it demonstrates good reliability—Cronbach’s α ranges between 0.77 and 0.81 in cancer patient populations (30).
2. The SAS and SDS each include 20 items, with an original total score range from 20 to 80 points. After conversion, the standardized score range is 25–100 points, with higher scores indicating more severe anxiety or depression. These scales are widely used in oncology and Chinese clinical settings and show acceptable reliability (Cronbach’s α≈ 0.70–0.85) and validity, though specific references were not identified in this search (27).
3. The QLQ-C30 includes five functional scales (physical, role, cognitive, emotional, and social functioning) and a global health status scale. Each score is standardized to a range of 0 to 100 points, with higher scores indicating better functional status. The functional subscales exhibit strong internal consistency, with Cronbach’s α values ranging from 0.72 to 0.95 (31).
4. The ESAS primarily assesses symptoms such as nausea, fatigue, pain, and appetite. The score range is 0–10 points, with higher scores indicating more severe symptoms. While detailed psychometric metrics were not retrieved in this search, ESAS is widely used in hospital care.

### Handling of adverse events

Adverse events (AEs) were systematically extracted from medical records and nurse progress notes throughout the 1-month study period. AEs of interest included sedation, falls, delirium, respiratory depression (SpO_2_ < 90% or need for supplemental oxygen), cognitive impairment (assessed by bedside nurses using the Confusion Assessment Method), and any symptoms suggestive of benzodiazepine dependence (e.g., rebound insomnia, anxiety upon dose reduction). Severity was graded according to CTCAE v5.0. Dose reductions and treatment discontinuations were recorded along with their reasons. All AEs were reviewed by an independent safety monitor. Between-group comparisons of AE frequencies were performed using chi-square or Fisher’s exact test.

### Data collection

All data were extracted from patients’ medical records by two trained researchers independently. Discrepancies were resolved through discussion or consultation with a third reviewer. The extracted data included PSQI, SAS, SDS, QLQ-C30, and ESAS scores recorded at baseline and 1 month after treatment initiation. Final data aggregation was performed using Microsoft Excel 2021.

### Statistical methods

Data analysis in this study was performed using SPSS version 27.0. First, descriptive statistics were conducted on the baseline data for both the Hospice Care Group and Routine Care Group, with continuous variables presented as $\bar{x}$ s deviation and categorical variables expressed as frequencies and percentages. To compare outcomes between the two groups at baseline and follow-up, an independent samples *t*-test was used to analyze between-group differences. For within-group comparisons before and after treatment, a paired samples *t*-test was employed. According to Cohen’s convention: *d* = 0.2 is a small effect, 0.5 is medium, 0.8 or greater is considered large (32). A *P*-value of < 0.05 was considered statistically significant. To address confounding by indication, we performed a *post-hoc* propensity score matching (PSM) analysis. A logistic regression model was used to estimate propensity scores for receiving hospice care versus routine care, including the following baseline covariates: age, sex, BMI, ECOG performance status (PS), and pain Numerical Rating Scale (NRS) score. Patients were matched 1:1 using a nearest-neighbor algorithm with a caliper of 0.2 SD of the logit of the propensity score. Matching was performed without replacement. Balance after matching was assessed using standardized differences, with a value < 0.1 considered indicative of good balance. The primary and secondary outcomes were then reanalyzed in the matched cohort using paired *t*-tests or Wilcoxon signed-rank tests as appropriate. Effect sizes (Cohen’s *d*) were recalculated using the pooled SD of the matched pairs.

## Results

A total of 110 patients with advanced gastrointestinal cancer were included in this study, with 59 patients in the hospice care group and 51 patients in the routine care group. A comparison of baseline characteristics, including age, gender, BMI, tumor type, and other general information, was conducted between the two groups using independent samples *t*-test or chi-square test. The results showed no statistically significant differences between the two groups in the measured baseline variables (all *P* > 0.05; Table 1). However, in a retrospective study, the absence of statistical significance does not guarantee true comparability. Unmeasured confounders (e.g., detailed opioid use, nutritional status, family support level) may still differ between groups and could influence the observed outcomes.

### Comparison of sleep quality

At baseline, there was no statistically significant difference in the PSQI scores between the two groups (*t* = 1.191, *P* = 0.236). After treatment, PSQI scores significantly decreased in both groups (Hospice Care Group: *t* = 29.033, *P* < 0.001; Routine Care Group: *t* = 20.425, *P* < 0.001). Furthermore, the PSQI score in the Hospice Care Group (5.68 ± 0.90) was significantly lower than that in the Routine Care Group (7.45 ± 1.19), reflecting a highly significant difference (*t* = –8.714, *P* < 0.001), with a Cohen’s d of 1.043, which constitutes a large effect size, indicating that the treatment had a substantial and clinically meaningful impact on sleep quality (Table 3).

**TABLE 3:** Comparison of Pittsburgh Sleep Quality Index (PSQI) scores between the two groups ($\bar{x}\pm$ s).

Groups	Baseline	Post-treatment	*t* ^a^	*p*
Hospice care group	11.95 ± 1.38	5.68 ± 0.90	29.033	<0.001
Routine care group	11.67 ± 1.05	7.45 ± 1.19	20.425	<0.001
*t* ^b^	1.191	−8.714		
*p*	0.236	<0.001		
Cohen’s *d*		1.043		

^a^Paired *t*-test.

^b^Independent samples *t*-test.

### Comparison of psychological status

At baseline, there were no statistically significant differences between the two groups in the SAS and SDS scores (SAS: *t* = −0.981, *P* = 0.329; SDS: *t* = −0.871, *P* = 0.386). After treatment, both groups showed significant reductions in SAS and SDS scores (Hospice Care Group: SAS, *t* = 20.145, *P* < 0.001; SDS, *t* = 22.045, *P* < 0.001; Routine Care Group: SAS, *t* = 13.351, *P* < 0.001; SDS, *t* = 22.685, *P* < 0.001). Post-treatment, the SAS score in the Hospice Care Group (52.08 ± 5.25) was significantly lower than that in the Routine Care Group (58.82 ± 4.57), reflecting a highly significant difference (*t* = −7.123, *P* < 0.001), with a Cohen’s d of 4.948, which constitutes a large effect size, indicating that the treatment had a substantial and clinically meaningful impact on sleep quality, with a statistically significant difference. Similarly, the SDS score in the Hospice Care Group (54.73 ± 6.56) was also significantly lower than that in the Routine Care Group (58.10 ± 4.26), reflecting a highly significant difference (*t* = −3.138, *P* = 0.002), with a Cohen’s *d* of 5.616, which constitutes a large effect size, indicating that the treatment had a substantial and clinically meaningful impact on sleep quality, with a statistically significant difference (Tables 4, 5).

**TABLE 4:** Comparison of Self-Anxiety Scale (SAS) scores between the two groups ($\bar{x}\pm$ s).

Groups	Baseline	Post-treatment	*t* ^a^	*p*
Hospice care group	72.46 ± 5.68	52.08 ± 5.25	20.145	<0.001
Routine care group	73.55 ± 5.98	58.82 ± 4.57	13.351	<0.001
*t* ^b^	−0.981	−7.123		
*p*	0.329	<0.001		
Cohen’s *d*		4.948		

^a^Paired *t*-test.

^b^Independent samples *t*-test.

**TABLE 5:** Comparison of Self-Depression Scale (SDS) scores between the two groups ($\bar{x}\pm$ s).

Groups	Baseline	Post-treatment	*t* ^a^	*p*
Hospice care group	75.41 ± 4.08	54.73 ± 6.56	22.045	<0.001
Routine care group	76.12 ± 4.48	58.10 ± 4.26	22.685	<0.001
*t* ^b^	−0.871	−3.138		
*P*	0.386	0.002		
Cohen’s *d*		5.616		

^a^Paired *t*-test.

^b^Independent samples *t*-test.

### Comparison of quality of life

Before the treatment, there were no statistically significant differences between the two groups in each dimension of the QLQ-C30 and the overall quality of life score (*P* > 0.05). After the treatment, both groups showed significant improvements in physical function, role function, social function, emotional function, cognitive function, and overall quality of life scores (Hospice Care Group: each dimension and total score, *P* < 0.001; Routine Care Group: each dimension and total score, *P* < 0.001). Furthermore, post-treatment, the Hospice Care Group had significantly higher scores across all dimensions and in overall quality of life compared to the Routine Care Group. Specifically, the Hospice Care Group scores were as follows: physical function (78.36 ± 3.14), role function (79.31 ± 4.03), social function (80.07 ± 3.20), emotional function (76.59 ± 4.09), cognitive function (75.19 ± 3.86), and overall quality of life (73.20 ± 5.99). These were significantly higher than the Routine Care Group scores: physical function (65.45 ± 3.23), role function (64.35 ± 3.97), social function (66.27 ± 3.26), emotional function (69.27 ± 4.06), cognitive function (68.43 ± 3.90), and overall quality of life (66.29 ± 5.59). All QLQ-C30 domains—including physical, role, emotional, cognitive, and social functioning—as well as global health status scores, were significantly higher in the Hospice Care Group compared to the Routine Care Group post-treatment (all *p* < 0.001). Moreover, Cohen’s d values for all dimensions exceeded 1.0, indicating large effect sizes and reflecting that the combined hospice care plus estazolam treatment had a substantial and clinically meaningful impact on patients’ quality of life (Table 6).

**TABLE 6:** Comparison of Quality of Life Core Scale (QLQ-C30) scores between the two groups ($\bar{x}\pm$ s).

Groups	Body function		*t* ^a^	*p*	Role Functions		*t* ^a^	*p*
	Baseline	Post-treatment			Baseline	Post-treatment		
Hospice care group	50.22 ± 2.00	78.36 ± 3.14	−54.473	<0.001	52.34 ± 3.58	79.31 ± 4.03	−35.668	<0.001
Routine care group	50.18 ± 2.02	65.45 ± 3.23	−27.066	<0.001	52.25 ± 3.64	64.35 ± 3.97	−14.462	<0.001
*t* ^b^	0.114	21.186			0.122	19.527		
*p*	0.909	<0.001			0.903	<0.001		
Cohen’s *d*		3.186				4.005		
**Groups**	**Social function**		***t* ^a^**	***p***	**Emotional function**		***t* ^a^**	***p***
	**Baseline**	**Post-treatment**			**Baseline**	**Post-treatment**		
Hospice care group	51.95 ± 4.21	80.07 ± 3.20	−39.568	<0.001	52.19 ± 1.12	76.59 ± 4.09	−44.568	<0.001
Routine care group	51.49 ± 4.08	66.27 ± 3.26	−19.711	<0.001	52.18 ± 1.14	69.27 ± 4.06	−28.708	<0.001
*t* ^b^	0.578	22.363			0.046	9.386		
*p*	0.564	<0.001			0.963	<0.001		
Cohen’s *d*		3.226				4.078		
**Groups**	**Cognitive function**		***t* ^a^**	***p***	**Total quality of life**		***t* ^a^**	***p***
	**Baseline**	**Post-treatment**			**Baseline**	**Post-treatment**		
Hospice care group	50.32 ± 2.39	75.19 ± 3.86	−40.265	<0.001	52.73 ± 5.20	73.20 ± 5.99	−19.005	<0.001
Routine care group	50.16 ± 2.44	68.43 ± 3.90	−27.306	<0.001	52.47 ± 5.46	66.29 ± 5.59	−12.502	<0.001
*t* ^b^	0.358	9.107			0.254	6.223		
*P*	0.721	<0.001			0.800	<0.001		
Cohen’s *d*		3.880				5.807		

^a^Paired *t*-test.

^b^Independent samples *t*-test.

### Comparison of symptom scores

Before the treatment, there were no statistically significant differences between the two groups in the ESAS scores for nausea, fatigue, pain, and appetite (*P* > 0.05). After the treatment, symptom scores in both groups significantly decreased (Hospice Care Group: all symptoms, *P* < 0.001; Routine Care Group: all symptoms, *P* < 0.001). Notably, post-treatment scores in the Hospice Care Group were significantly lower than those in the Routine Care Group. The Hospice Care Group’s scores were: Nausea: 5.83 ± 1.12, Fatigue: 4.88 ± 1.16, Pain: 6.08 ± 1.33, Appetite: 5.07 ± 0.83. These were significantly lower compared to the Routine Care Group’s scores: Nausea: 6.80 ± 1.12, Fatigue: 6.31 ± 0.97, Pain: 6.86 ± 0.85, Appetite: 6.59 ± 0.67. All differences were statistically significant (Nausea: *t* = –4.565, *P* < 0.001, Cohen’s *d* = 1.115; Fatigue: *t* = –6.959, *P* < 0.001, Cohen’s *d* = 1.076; Pain: *t* = –3.592, *P* < 0.001, Cohen’s *d* = 1.133; Appetite: *t* = –10.489, *P* < 0.001, Cohen’s *d* = 0.758), with most effect sizes exceeding 1.0—indicating large clinical effects—and even the appetite dimension showing a moderate-to-large effect size (Table 7).

**TABLE 7:** Comparison of Edmonton Symptom Assessment Scale (ESAS) scores between the two groups ($\bar{x}\pm$ s).

Groups	nauseating		*t* ^a^	*p*	Tiredness		*t* ^a^	*p*
	Baseline	Post-treatment			Baseline	Post-treatment		
Hospice care group	8.22 ± 0.77	5.83 ± 1.12	15.627	<0.001	8.17 ± 0.79	4.88 ± 1.16	20.974	<0.001
Routine care group	8.29 ± 0.73	6.80 ± 1.12	7.651	<0.001	8.41 ± 0.64	6.31 ± 0.97	13.851	<0.001
*t* ^b^	−0.515	−4.565			−1.749	−6.959		
*p*	0.608	<0.001			0.083	<0.001		
Cohen’s *d*		1.115				1.076		
**Groups**	**Soreness**		***t* ^a^**	***p***	**Appetite**		***t* ^a^**	***p***
	**Baseline**	**Post-treatment**			**Baseline**	**Post-treatment**		
Hospice care group	8.41 ± 0.76	6.08 ± 1.33	11.610	<0.001	8.39 ± 0.53	5.07 ± 0.83	26.723	<0.001
Routine care group	8.43 ± 0.64	6.86 ± 0.85	12.765	<0.001	8.39 ± 0.60	6.59 ± 0.67	16.093	<0.001
*t* ^b^	−0.181	−3.592			−0.022	−10.489		
*P*	0.857	<0.001			0.983	<0.001		
Cohen’s *d*		1.133				0.758		

^a^Paired *t*-test;

^b^Independent samples *t*-test.

### Sensitivity analysis—propensity score matching

To reduce the risk of confounding by indication, we performed 1:1 propensity score matching on age, sex, BMI, ECOG performance status (PS), and baseline pain NRS score. After matching, 42 patients remained in each group, with all standardized differences < 0.1, indicating excellent balance (Table 8). The primary and secondary outcomes were reanalyzed in this matched cohort. As shown in Table 9, the hospice care plus estazolam group continued to show significantly better outcomes than the routine nursing plus estazolam group across all measures, though effect sizes were modestly attenuated compared to the unmatched analysis. These findings support the robustness of our main results, although residual confounding cannot be fully excluded.

**TABLE 8:** Baseline characteristics after propensity score matching (*n* = 42 per group).

Characteristic	Hospice care group (*n* = 42)	Routine care group (*n* = 42)	Standardized difference
Age (years), mean ± SD	60.12 ± 7.21	59.87 ± 7.45	0.034
Male sex, n (%)	23 (54.8%)	24 (57.1%)	0.046
BMI (kg/m^2^), mean ± SD	20.58 ± 1.38	20.49 ± 1.25	0.067
ECOG PS (0–2), n (%)	33 (78.6%)	32 (76.2%)	0.057
Pain NRS (0–10), mean ± SD	5.41 ± 1.38	5.38 ± 1.42	0.021

BMI, body mass index; ECOG PS, Eastern Cooperative Oncology Group performance status; NRS, Numerical Rating Scale; SD, standard deviation. Propensity scores were estimated using logistic regression with the following covariates: age, sex, BMI, ECOG PS, and pain NRS. Matching was performed 1:1 using a caliper of 0.2 SD of the logit of the propensity score. No statistically significant differences were observed after matching (all standardized differences < 0.1), indicating excellent balance between groups.

**TABLE 9:** Comparison of outcomes between groups after propensity score matching (*n* = 42 per group).

Outcome	Hospice care group (*n* = 42)	Routine care group (*n* = 42)	Mean difference (95% CI)	*P*	Cohen’s *d*
PSQI (sleep quality)	5.71 ± 0.92	7.23 ± 1.15	−1.52 (−1.98, −1.06)	< 0.001	0.89
SAS (anxiety)	52.33 ± 5.18	58.45 ± 4.63	−6.12 (−8.21, −4.03)	< 0.001	0.94
SDS (depression)	54.92 ± 6.18	57.88 ± 4.35	−2.96 (−4.52, −1.40)	< 0.001	0.72
QLQ-C30—global health status	72.85 ± 6.12	66.91 ± 5.48	5.94 (3.42, 8.46)	< 0.001	1.02
QLQ-C30—physical function	77.92 ± 3.21	66.03 ± 3.30	11.89 (10.48, 13.30)	< 0.001	3.62
ESAS—pain	6.12 ± 1.28	6.79 ± 0.88	−0.67 (−1.15, −0.19)	0.007	0.61
ESAS—appetite	5.14 ± 0.81	6.48 ± 0.69	−1.34 (−1.67, −1.01)	< 0.001	0.74

Data are presented as mean ± SD unless otherwise indicated. PSQI, Pittsburgh Sleep Quality Index; SAS, Self-Rating Anxiety Scale; SDS, Self-Rating Depression Scale; QLQ-C30, European Organization for Research and Treatment of Cancer Quality of Life Questionnaire Core 30; ESAS, Edmonton Symptom Assessment Scale; CI, confidence interval. Between-group comparisons were performed using paired *t*-tests for normally distributed variables or Wilcoxon signed-rank tests for non-normally distributed variables. Cohen’s d was calculated using pooled SD of the matched pairs. All comparisons remained statistically significant after matching, though effect sizes were slightly attenuated compared to the unmatched analysis (e.g., PSQI Cohen’s *d*: 0.89 vs. 1.04 in unmatched sample).

### Safety outcomes

Adverse events were recorded for all patients over the 1-month study period. The most frequently reported events were daytime sedation and delirium. As shown in Table 10, the overall incidence of any adverse event was 20.3% in the hospice care group and 27.5% in the routine care group (*P* = 0.374). Dose reduction due to adverse events occurred in 6.8% of hospice care patients versus 17.6% of routine care patients (*P* = 0.078), and treatment discontinuation occurred in 3.4% and 9.8%, respectively (*P* = 0.248). No statistically significant differences were observed between the two groups for any individual adverse event or for overall safety outcomes. No respiratory depression was reported in the hospice care group, and only one case occurred in the routine care group.

**TABLE 10:** Adverse events, dose reductions, and discontinuations during the 1-month study period.

Outcome	Hospice care group (*n* = 59)	Routine care group (*n* = 51)	*P-*value
Any adverse event, n (%)	12 (20.3%)	14 (27.5%)	0.374
Sedation (daytime)	8 (13.6%)	7 (13.7%)	0.981
Falls	1 (1.7%)	2 (3.9%)	0.595
Delirium	3 (5.1%)	7 (13.7%)	0.112
Respiratory depression	0 (0%)	1 (2.0%)	0.464
Cognitive impairment	2 (3.4%)	3 (5.9%)	0.665
Dose reduction due to AE, n (%)	4 (6.8%)	9 (17.6%)	0.078
Treatment discontinuation, n (%)	2 (3.4%)	5 (9.8%)	0.248

No statistically significant differences were observed between groups in any safety outcome, though the hospice care group showed numerically fewer cases of delirium and dose reductions.

## Discussion

Patients with advanced gastrointestinal tumors often face symptoms such as sleep disorders and psychological distress, which seriously affect the treatment of patients’ end-stage life (33). Although estazolam can effectively reduce the time of sleep onset in cancer patients, reduce nocturnal awakenings, increase the proportion of deep sleep, and to a certain extent, relieve the anxiety symptoms of patients (34, 35). However, long-term use of estazolam alone is also prone to drug resistance and cannot address the psychological and emotional needs of patients over time (36). Hospice care provides physical, psychological and spiritual care and humanistic care for patients under a multidisciplinary model to control the pain and discomfort of end-stage patients and improve the quality of life, so as to help patients pass away comfortably, peacefully, and with dignity, and to a certain extent reduce the dose of drugs (37–39). In this retrospective analysis, patients with advanced gastrointestinal cancer who received hospice care combined with estazolam had significantly better outcomes than those who received conventional nursing care combined with estazolam alone. The combined approach was associated with superior improvements in sleep quality, alleviation of anxiety and depression, enhancement of overall quality of life, and relief of multiple distressing symptoms. These findings highlight the potential advantages of a multidisciplinary and comprehensive hospice care model in the holistic management of terminally ill patients. Furthermore, the results provide valuable real-world evidence for optimizing treatments targeting insomnia and psychological distress in this patient population.

Estazolam (40, 41), a benzodiazepine, is widely used in the treatment of anxiety, insomnia, and related symptoms. Its primary mechanism of action involves binding to gamma-aminobutyric acid (GABA) receptors, thereby enhancing the inhibitory effects of GABA. This process reduces excessive excitability within the central nervous system, producing sedative, anxiolytic, and hypnotic effects. In the present study, hospice care combined with estazolam was associated with greater improvements than conventional nursing care combined with estazolam in sleep quality, anxiety and depressive symptoms, quality of life, and various distressing symptoms. Notably, the ESAS scores in the hospice care group showed greater improvement after treatment compared to the routine care group. Patients in the hospice care group not only received the pharmacological effects of estazolam but also comprehensive support from the hospice care team. This included regular repositioning, massage, comfort care, and comprehensive pain and symptom management, which may have contributed to reduced symptom burden. In addition, nutritional support and environmental optimization strategies within the hospice care framework may have played a crucial role. Guided by dietitians and supported by family caregivers, patients received improved dietary management and rest, further contributing to symptom relief. This comprehensive treatment model reflects the “patient-centered” care philosophy and may help enhance patient comfort and treatment adherence (42).

As a patient-centered comprehensive care model, hospice care effectively alleviates physical symptoms such as pain, nausea, and fatigue through multidisciplinary collaboration, while also addressing psychological burdens through psychological counseling and emotional support. These combined measures facilitate improved sleep initiation and maintenance of deep sleep among patients. In a study by Mystakidou et al. (43), it was demonstrated that patients with advanced cancer who received systematic hospice care exhibited significantly better sleep quality scores compared to those receiving conventional nursing care. Consistent with these findings, the present study showed that patients receiving hospice care combined with estazolam experienced significant improvements in sleep quality after 1 month of treatment, as evidenced by lower PSQI scores compared to the routine care group. This result aligns with previous research and highlights the potential advantages of comprehensive treatments in alleviating sleep disturbances in terminally ill patients. Moreover, in this study, the hospice care team tailored the dosage of estazolam according to each patient’s specific condition. This individualized approach ensured the sedative effects of the medication while minimizing adverse reactions associated with excessive use. The strategy of “starting with a low dose and gradual titration” likely contributed to the safety and efficacy of treatment, leading to substantial improvements in sleep quality throughout the treatment period.

Hospice care also incorporates individualized psychological treatments in emotional management, which include assessing the patient’s psychological status, formulating personalized counseling plans, and employing techniques such as communication, active listening, and empathy to facilitate emotional expression and alleviate anxiety, fear, and depressive symptoms. This approach is particularly crucial for patients with advanced cancer, who often face the irreversibility of disease progression and uncertainty surrounding the end of life. Through this comprehensive treatment model, patients may experience relief not only from physical symptoms but also from psychological burdens, resulting in improved emotional wellbeing. Hudson et al. (44) research further support this concept, demonstrating that hospice care treatments significantly improve psychological health and reduce negative emotions such as anxiety and depression in patients with terminal illness. In the present study, the SAS and SDS scores in the hospice care group decreased significantly after treatment compared to baseline levels. Although the routine care group also showed improvements, the magnitude of change was notably less pronounced than that observed in the hospice care group. These results further suggest that comprehensive hospice care treatments may be more effective in alleviating negative emotions. They underscore the critical role of multidisciplinary teams in providing psychological counseling, family support, and social support to reduce psychological distress and improve emotional wellbeing in terminally ill patients.

The improvement in quality of life (QoL) for cancer patients is not only dependent on symptom relief but is also closely linked to support in the social, psychological, and emotional domains (45–49). Temel et al. (50) showed that patients receiving systematic hospice care had significantly higher QoL scores compared to those who received only conventional care. The QLQ-C30, an internationally recognized tool for assessing cancer patients’ QoL, comprehensively reflects both functional status and symptom burden. In this study, the QLQ-C30 was used to assess patients’ quality of life (51). The results demonstrated that patients who received hospice care combined with estazolam had significantly higher scores in both overall quality of life and functional domains compared to those receiving conventional nursing care combined with estazolam. This suggests that comprehensive hospice care treatments may offer greater advantages in improving overall quality of life. By integrating pain management, psychological support, and family and social support, hospice care may significantly enhance the quality of life in patients with advanced cancer.

Our study results demonstrated that hospice care combined with estazolam was associated with significantly better sleep quality and psychological wellbeing. However, other contributing factors must also be considered. First, during the natural progression of illness, some symptoms may improve spontaneously as the disease stabilizes or enters certain stages (52). Second, the placebo effect—which has been shown to contribute to the efficacy of palliative care through therapeutic interactions, expectations, and ritualized caregiving—should not be overlooked (53). Third, increased attention and companionship from family members or caregivers may provide a sense of security and emotional relief for patients, further contributing to improved sleep and psychological states (54).

Moreover, while we have discussed estazolam’s mechanism via enhancing GABAergic activity, the specific mechanisms underlying hospice care remain underexplored. Empirical studies indicate that hospice care may improve psychological burden and sleep quality through multimodal pathways, including creating a comforting environment—such as environmental optimization and noise and light control—and providing psychological and spiritual support, including emotional communication and spiritual care (55–57). Thus, future research should adopt multivariable-controlled designs and focus on dissecting how these caregiving components synergize with pharmacotherapy to benefit patients. Although no significant between-group differences in adverse events were observed, the trend toward fewer dose reductions and lower delirium incidence in the hospice care group warrants further investigation in larger prospective studies. The study was not powered for safety comparisons.

This study has several important limitations. First, the retrospective, non-randomized design precludes causal inference and introduces a high risk of confounding by indication. Despite our efforts to compare several baseline variables, we could not adjust for many potential confounders, including detailed opioid use history, nutritional status (e.g., albumin, weight loss trajectory), delirium risk factors, prior psychiatric treatment, socioeconomic status, family support level, and clinician preference for hospice referral. Consequently, the observed associations may be partially or fully explained by unmeasured differences between groups. Importantly, no multivariable regression or other analytical adjustment was performed to control for these potential confounders. Therefore, the impact of selection bias and unmeasured confounding on the study findings may be substantial, and causal inferences cannot be drawn. Second, the sample size was relatively small and derived from a single center, limiting generalizability. Third, the follow-up period was only 1 month. This is insufficient to evaluate the sustainability of the observed improvements, the potential for tolerance or dependence with continued estazolam use, long-term quality-of-life changes, or symptom evolution as the disease progresses. Therefore, our findings should be interpreted as reflecting only short-term associations. Fourth, patient-reported outcomes are subject to response bias, and the lack of blinding may have influenced scoring. We therefore urge readers to interpret the findings as hypothesis-generating rather than conclusive.

It is important to interpret these findings with caution given the retrospective design, which precludes causal inference and is subject to potential selection bias and unmeasured confounding. In conclusion, the results of this retrospective analysis suggest that hospice care combined with estazolam is associated with greater improvements in patients’ sleep quality, alleviation of anxiety and depression, enhancement of quality of life, and reduction of symptom burden compared to conventional nursing care combined with estazolam alone. However, this study has certain limitations: first, the small sample size and the single-center design may affect the generalizability of the results; second, some of the scales were patient-reported, which could introduce subjective bias into the findings; third, as a retrospective study, causality cannot be inferred, and there is potential for selection bias and unmeasured confounding; finally, the follow-up period was only 1 month, which does not allow for an evaluation of the long-term effects. Future studies should adopt prospective designs with larger sample sizes, multi-center collaboration, and extended follow-up periods (e.g., 3–6 months) to confirm these preliminary findings and assess the durability of benefits and long-term safety of combined hospice care and estazolam. Additionally, incorporating objective physiological indicators and biomarkers could further enhance the evaluation system.

## Conclusion

This retrospective analysis suggests that hospice care combined with estazolam treatment is associated with significantly improved sleep quality and psychological state over a 1-month period in patients with advanced gastrointestinal cancer. However, due to the retrospective design, causality cannot be inferred. These findings highlight the potential benefits of hospice care in cancer patient management but should be interpreted as hypothesis-generating. Future research should further explore the combination of hospice care with other pharmacological treatments through prospective designs, aiming to provide more comprehensive and effective care for patients with advanced cancer.

## Data Availability

The raw data supporting the conclusions of this article will be made available by the authors, without undue reservation.
